# Supplementary material for: Intentions of Canadian health professionals towards recommending exercise for people living with ALS
Source: BMC Neurol. 2019 Aug 22;19:204. doi: 10.1186/s12883-019-1426-z (PMC6706893; doi:10.1186/s12883-019-1426-z)
Supplement: Supplementary file 1 — Supplementary Perceived Behavioral Control: individual items. (PDF 16 kb) [file 12883_2019_1426_MOESM1_ESM.pdf]

Table 5 *Supplementary* Perceived Behavioral Control: individual items

	Strength	Aerobic	Flexibility
It is easy to provide exercise prescriptions for pALS.			
Physicians	4.0±1.9	4.8±1.9	4.8±1.5
HP_Yes	4.4±1.7	4.3±1.6	5.6±1.3
I am confident in providing exercise counsel to pALS			
Physicians	4.8±1.7	5.0±1.5	5.4±1.5
HP_Yes	4.9±1.6	5.2±1.6	5.5±1.9
My clinic accommodates exercise advise for pALS (i.e. has the facility, space, equipment)			
Physicians	3.0±2.1	2.9±2.2	3.6±2.5
HP_Yes	3.8±2.1	4.0±2.0	4.3±2.1
My clinic encourages exercise counsel for pALS			
Physicians	4.4±2.0	4.8±1.8	5.9±1.1
HP_Yes	4.9±1.8	5.2±1.7	5.9±1.5
